# CRISPR-Cas9 Knockdown and Induced Expression of CD133 Reveal Essential Roles in Melanoma Invasion and Metastasis

**DOI:** 10.3390/cancers11101490

**Published:** 2019-10-03

**Authors:** Cynthia M. Simbulan-Rosenthal, Ryan Dougherty, Sahar Vakili, Alexandra M. Ferraro, Li-Wei Kuo, Ryyan Alobaidi, Leala Aljehane, Anirudh Gaur, Peter Sykora, Eric Glasgow, Seema Agarwal, Dean S. Rosenthal

**Affiliations:** 1 Department of Biochemistry and Molecular & Cellular Biology, Georgetown University School of Medicine, Washington, DC 20007, USA; simbulac@georgetown.edu (C.M.S.-R.); rdd40@georgetown.edu (R.D.); sv457@georgetown.edu (S.V.); amf315@georgetown.edu (A.M.F.); lk702@georgetown.edu (L.-W.K.); raa125@georgetown.edu (R.A.); la591@georgetown.edu (L.A.); ag854@georgetown.edu (A.G.); 2 Amelia Technologies, Rockville, MD 20850, USA; peters@ameliatechnologies.com; 3 Department of Oncology, Georgetown University School of Medicine, Washington, DC 20007, USA; eg239@georgetown.edu; 4 Department of Pathology, Georgetown University School of Medicine, Washington, DC 20007, USA; seema.agarwal@georgetown.edu

**Keywords:** melanoma initiating cells, invasion, metastasis, matrix metalloproteinases, CRISPR-Cas9 knockdown

## Abstract

CD133, known as prominin1, is a penta-span transmembrane glycoprotein presumably a cancer stem cell marker for carcinomas, glioblastomas, and melanomas. We showed that CD133(+) ‘melanoma-initiating cells’ are associated with chemoresistance, contributing to poor patient outcome. The current study investigates the role(s) of CD133 in invasion and metastasis. Magnetic-activated cell sorting of a melanoma cell line (BAKP) followed by transwell invasion assays revealed that CD133(+) cells are significantly more invasive than CD133(−) cells. Conditional reprogramming of BAKP CD133(+) cells maintained stable CD133 overexpression (BAK-R), and induced cancer stem cell markers, melanosphere formation, and chemoresistance to kinase inhibitors. BAK-R cells showed upregulated CD133 expression, and consequently were more invasive and metastatic than BAK-P cells in transwell and zebrafish assays. CD133 knockdown by siRNA or CRISPR-Cas9 (BAK-R-T3) in BAK-R cells reduced invasion and levels of matrix metalloproteinases MMP2/MMP9. BAK-R-SC cells, but not BAK-R-T3, were metastatic in zebrafish. While CD133 knockdown by siRNA or CRISPR-Cas9 in BAK-P cells attenuated invasion and diminished MMP2/MMP9 levels, doxycycline-induced CD133 expression in BAK-P cells enhanced invasion and MMP2/MMP9 concentrations. CD133 may therefore play an essential role in invasion and metastasis via upregulation of MMP2/MMP9, leading to tumor progression, and represents an attractive target for intervention in melanoma.

## 1. Introduction

Melanoma is a deadly form of cancer, with 23,000 cases and 7320 deaths in the United States. In 16 other countries such as Australia and New Zealand, melanoma incidence and death rates are even higher [1]. As with other cancers, patient survival decreases with progression of the disease. Invasion and metastasis, hallmarks of progression, are therefore key factors that determine clinical outcomes. Not surprisingly, there has been a concerted effort to determine genes responsible for progression. Metastasis inhibitor(s) such as *Kiss1* [2] and others located on chromosome *6p* [3], as well as inducers of melanoma metastasis such as BMI1 [4] have been investigated. Some genes alter the course of early stages of tumorigenesis along with metastasis, while others exert their effects on progression alone [5]. Some inducers of metastasis, such as BMI, also induce sets of genes that generate a cancer stem cell phenotype [4], indicating a connection between stemness and cancer progression. 

One of the most commonly-used markers for stem cells for a number of cancers is CD133, known as prominin1 (PROM1), a pentaspan transmembrane glycoprotein also expressed in presumptive stem cells of some normal tissues. CD133 is believed to be a stem cell marker for normal hematopoietic cells [6,7], endothelial cells, neuronal and glial cells [6], as well as cells from adult kidney, mammary gland, trachea, salivary gland, uterus, placenta, digestive tract, testes, epidermal [8], and intestinal stem cells [9,10,11,12]. The importance of CD133 in retinal development has been shown in mouse knockout models, as well as in human genetic disorders in which mutations and deletions are associated with retinitis pigmentosa and macular degeneration [13,14,15]. 

CD133 is expressed in cancer stem cells isolated from cancers, including those of the brain [16,17] ovary [18], liver [19] prostate [20] pancreas [21], and colon [22,23], and in melanomas [24]. Several properties define stem cells, including potency and self-renewal; for cancer stem cells this latter property is assayed by the ability to serially propagate tumors in immunocompromised mice [24,25,26,27]. The existence of melanoma stem cells can be model-specific [28], and support the idea that melanomas possess microenvironment-regulated phenotypic plasticity [29,30,31,32], resulting in the use of a less controversial term ‘melanoma-initiating cells’ (MIC). In any case, we, along with others, have shown that CD133(+) MIC are associated with drug resistance [33]. Because of these characteristics, CD133(+) MIC [34] and other cancer stem cells [35] have been proposed to play a critical role in recurrence and reduced survival, and are of interest as an anti-cancer vaccination component, with some success in mouse models of melanoma [36]. 

In order for cells to form metastases, they must be able to detach from the primary tumor site, intravasate, and survive in lymphatic or blood vessels to disperse to other sites, extravasate, and attach at distant sites, and to interact with and modify their new microenvironment in order to survive and proliferate. For invasion, one important set of enzymes include those responsible for remodeling primary and metastatic sites. Upregulation of matrix metalloproteinases (MMPs), especially MMP2 and MMP9, appears to be particularly important in melanoma invasiveness [37,38,39]. A key role for MMP9 was demonstrated in studies that showed that this protease promoted melanoma invasiveness by degrading components of the extracellular matrix [40,41,42,43]. MMP9 expression is regulated by several pathways and epigenetic alterations [44,45,46]; overexpression can be the result of aberrant activation of the MAPK and AKT/mTOR signaling pathways almost always found in melanoma [47,48]. MMP9 expression is also regulated by several miRNAs [49,50].

For later stages of metastasis, the pathways are not as clear. In many cases, this process is related to expression of attachment and survival proteins. Together, invasion and metastasis, in concert with drug and immune resistance, determine the progression of the tumor, and ultimately, the survival of the patient. With the recent introduction of the immune checkpoint inhibitors and selective tyrosine kinase inhibitors, including BRAF and MEK inhibitors, there has been a significant improvement in the progression-free survival (PFS) and overall survival (OS) of patients with melanoma [51,52]. However, many patients develop resistance, significantly reducing their response to these therapeutics [39,53]. The time it takes to develop resistance is particularly abbreviated, due in part to the markedly high mutation rate of cutaneous melanomas compared to nearly all other solid tumors [54,55,56]. As a result, the cutaneous melanoma mortality rate is 2.2 to 2.7 per 100,000 [57,58,59]. 

Cutaneous melanoma is associated with driver mutations in the MAPK pathway, along with changes in PI3K/Akt/mTOR, p16/CDK4Rb, Wnt, and/or p53 [60,61]. *BRAF*-mutant melanoma can be treated with targeted therapy using mutant BRAF and MEK inhibitors in combination. MEK inhibition can also be effective for NRAS-mutated melanomas in vitro [62], as well as in vivo [63]. Anti–PD-1, anti-PD-L1, and anti-CTLA4 immunotherapies are sometimes efficacious for patients with *NRAS* metastatic tumors, but unfortunately the majority of patients are not responsive, in which case carboplatin, dacarbazine (Dimethyl Triazeno Imidazol Carboxamide; DTIC), or temozolomide (TMZ) are added as auxiliary treatments with limited success [64]. 

We recently showed that CD133 immunopositivity is correlated with recurrent patient disease, poor clinical outcomes, and decreased overall survival [34]. Furthermore, a query of the TCGA database with the UCSC Xena Functional Genomics Explorer (https://xenabrowser.net) showed a significant negative correlation between CD133 expression and days to death (Kaplan-Meier Estimator) [34]. We also showed that CD133(+) cells isolated from patient tumors formed tumors in nude mouse xenografts, whereas CD133(−) cells did not [34]. In the current study, we analyzed the potential roles of CD133 in tumorigenesis, invasion, and metastasis. CD133(+) cells showed increased invasion and metastasis following MACS sorting and conditional reprogramming. siRNA and CRISPR-Cas9 knockdown of CD133 reverted this phenotype in several different patient-derived melanoma lines. Further, doxycycline-inducible expression of CD133 increased melanoma invasion and levels of endogenous and secreted MMP9. This suggests that CD133 plays an essential role in melanoma invasion and metastasis, and is an attractive target for intervention.

## 2. Results

### 2.1. Magnetically-Sorted (MACS) CD133(+) Melanoma Cells Survive in Xenografts and Give Rise to Tumors in Mixed Populations, But Require Reprogramming to Maintain CD133 Expression in Culture

**Figure 1:**
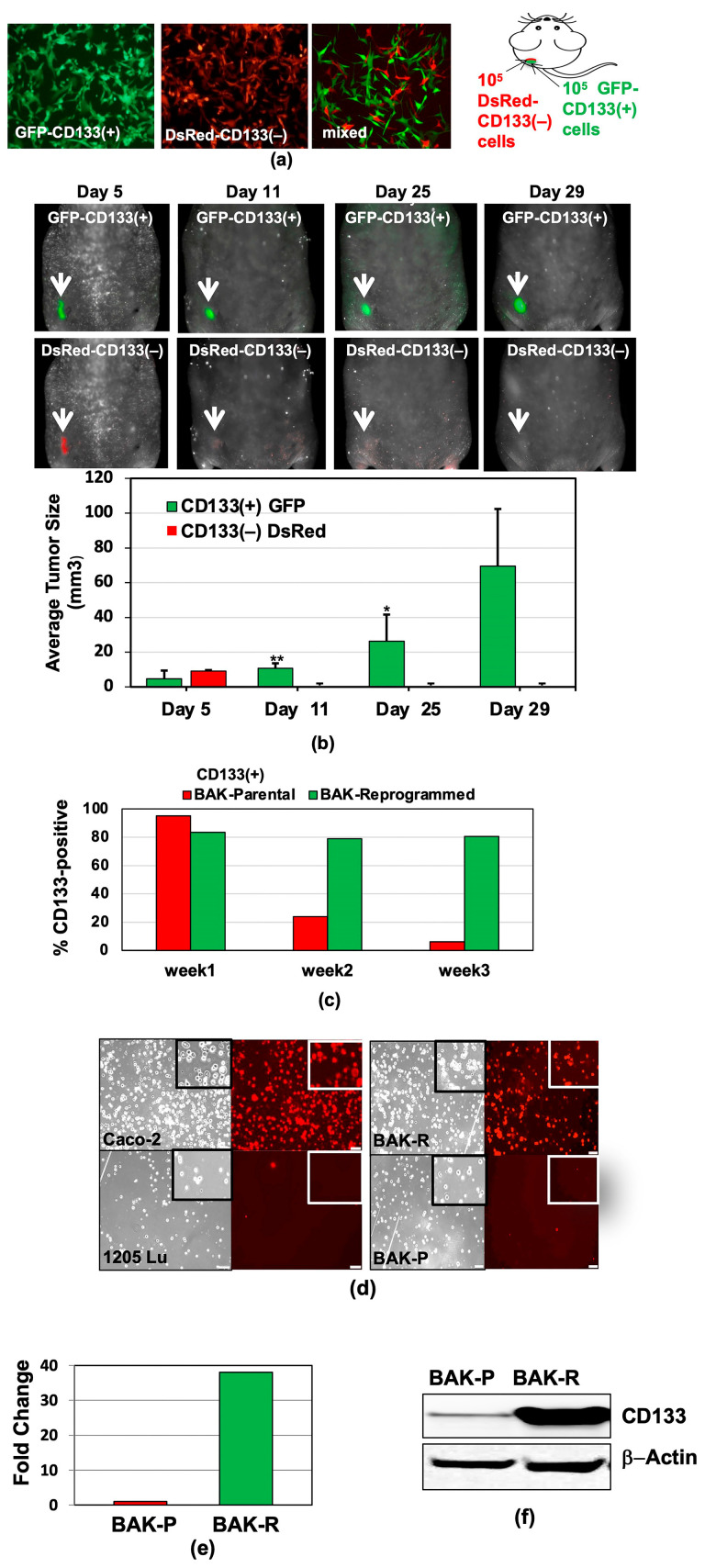
Preferential survival and growth of MACS-sorted CD133(+) cells in mixed population xenografts, and sustained CD133 expression after reprogramming. GFP-CD133(+) and DsRed-CD133(−) cells were isolated by MACS (**a**), injected into the flanks of nude mice, and tumors were visualized and measured by Maestro in vivo imaging after indicated periods of time. *p*-values of <0.05 were considered statistically significant and represented with a single asterisk, while *p* < 0.01 are labeled with two asterisks (**b**). (**c**) CD133 expression of reprogrammed BAK-R MIC and BAK-P populations assessed by immunofluorescent staining with anti-CD133-PE for 3 weeks show that BAK-R, but not BAK-P melanoma cells, exhibit sustained CD133 expression. Representative images after immunofluorescent staining of BAK-P and BAK-R cells with anti-CD133-PE ((**d**), right panels) as well as qRT-PCR (**e**) and immunoblot (**f**) analyses showing elevated CD133 expression in BAK-R compared to BAK-P cells. CD133(+) Caco-2 colon cancer cells and CD133(−) 1205Lu melanoma cells served as positive and negative controls, respectively ((**d**); left panels).

BRAF^WT^/NRAS^Q61K^ melanoma cells were derived from a patient (BAK parental (BAK-P) cell line), and separated by magnetic-activated cell sorting (MACS) into CD133(+) and CD133(−) subpopulations. Immunofluorescent staining confirmed CD133 positivity to be >85% in the CD133(+) BAK-P and less than 5% in the CD133(−) cells, confirming our previous results [33]. The tumor-initiating ability of the CD133(+) BAK-P was examined in an in vivo mouse xenograft model. After MACS sorting, as few as 1000 CD133(+) melanoma cells, but not CD133(−) cells, formed tumors by 7 weeks [33]. To track the subpopulations, early passages (<20; 40 population doublings) of BAK-P cells were stably transduced with DsRed or GFP prior to MACS separation into CD133(+) and CD133(−) subpopulations. Equal numbers of GFP-CD133(+) (left) and DsRed-CD133(−) (middle) melanoma cells were then mixed (Figure 1a) and subsequently subcutaneously injected into athymic nude mice; tumor growth was monitored over 8 weeks using a Maestro^TM^ in vivo fluorescent imaging system (CRI, Inc., Hopkinton, MA, USA). To determine the fate of each cell type in the mixed population, a shorter time course was performed. DsRed-CD133(−) cells were visible 5 days after xenografting, but were not detectable by 11 days, suggesting that CD133-positivity is correlated with the ability to survive for prolonged periods of time in the xenograft environment, allowing cells to form tumors weeks later. Interestingly, whereas the DsRed-CD133(−) population (lower panels) was no longer visible in the tumor after several days, the GFP-CD133(+) population (upper panels) survived, expanded, and contributed to tumor formation (Figure 1b). Furthermore, this survival is not conferred upon DsRed-CD133(−) cells either by cell contact or by paracrine factors released by GFP-CD133(+) cells. Finally, it appears that the CD133(−) phenotype is somewhat stable in this model, since early and late tumors all express GFP, and CD133(−) cells do not appear to convert to a tumorigenic phenotype, since no DsRed is seen in any tumors.

While CD133(+) cells were somewhat phenotypically stable in vivo, when MACS-sorted CD133(+) BAK cells were maintained in 2D culture, a marked loss of CD133 expression was noted by 2 weeks in culture (Figure 1c). We therefore determined if we could somehow capture a stable population of CD133(+) cells. To this end, CD133(+) cells were isolated by MACS and subjected to conditional reprogramming using the “Georgetown Method” [65,66]. Cells were maintained under Conditionally Reprogrammed Cell (CRC) protocol conditions using CRC medium (DMEM-F12/FY) containing the ROCK inhibitor Y27632, as described in Materials and Methods [65,66]; this resulted in a population that was stably ~80% CD133(+), even in 2D culture (Figure 1c,d). Quantitative RT-PCR (qRT-PCR; Figure 1e) and immunoblot analyses (Figure 1f) further verified elevated CD133 expression at the RNA and protein levels in the BAK reprogrammed cells (BAK-R) relative to the BAK parental population (BAK-P) from which they were derived.

### 2.2. MACS-Sorted BAK-P CD133(+) Melanoma Cells and CRC BAK-R Cells Both Exhibit Markers of Cancer Stem Cells, Form Melanospheres, and Are Resistant to Kinase Inhibitors

**Figure 2:**
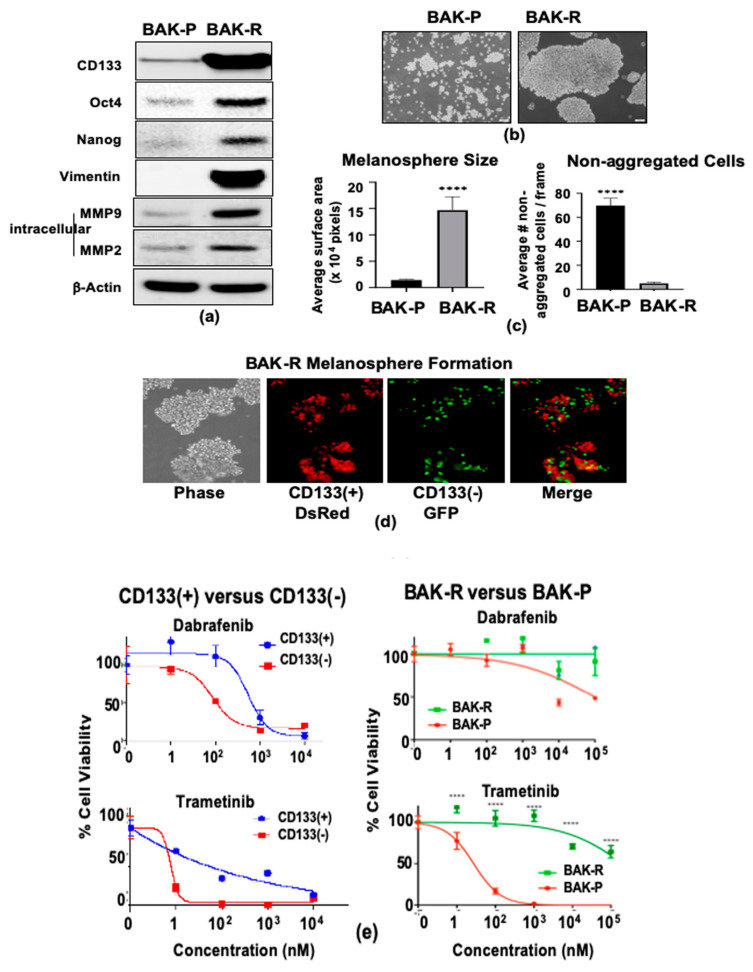
Compared to BAK-P, BAK-R cells strongly express markers of cancer stem cells (Oct4, Nanog), EMT (vimentin), and invasion (MMP2 and MMP9) as shown by immunoblot analysis (**a**), melanosphere formation (**b**–**d**) and resistance to kinase inhibitors (**e**). BAK-P and BAK-R cells were cultured separately in melanosphere conditions, imaged (**b**) and melanosphere sizes as well as average number of non-aggregated cells were quantified by Image J analysis (**c**). CD133(+) DsRed and CD133(−) GFP BAK-R cells were recombined in a 1:1 ratio and grown in low attachment plates (**d**). CD133(+) cells derived by MACS sorting (left column) and BAK-R (right column) both exhibit increased resistance to dabrafenib (top) or trametinib (bottom), compared to CD133(−) cells (left panel) or parental cells (BAK-P; right panel). *p*-values < 0.0001 are shown as four asterisks (**c**,**e**).

Cancer stem cell properties of MACS-sorted CD133(+) BAK-P melanoma were previously verified by immunofluorescent staining and qRT-PCR analysis for stem cell markers, including Oct 3/4, Nestin, and ABCB5 [33]. Immunoblot analysis revealed strong CD133-positivity of BAK-R compared to BAK-P cells, as well as increased expression of the stem cell markers Oct4 and Nanog (Figure 2a). Vimentin and matrix metalloproteinases-9 and 2 (MMP9 and MMP2), markers of epithelial–mesenchymal transition (EMT) and invasion, respectively, are also markedly upregulated in BAK-R cells. 

Other hallmarks of MICs include the ability to form large melanospheres and resistance to chemotherapeutic drugs (MAPK inhibitors). When cultured in 3D melanosphere conditions, BAK-P cells, composed primarily of CD133(−) cells, grew mostly as single dispersed cells and were only able to form small melanospheres, whereas BAK-R cells were found in huge melanospheres (Figure 2b,c). BAK-R cells were stably transduced with DsRed or GFP expression vectors, and magnetically sorted into CD133(+) and CD133(−) subpopulations. CD133(+) DsRed and CD133(−) GFP BAK-R cells were then mixed in equal numbers, and cultured under melanosphere conditions. Interestingly, while CD133(+) DsRed BAKR cells aggregated within the large melanospheres, CD133(−) GFP cells did not appear to self-associate, and were scattered throughout the larger mixed-cell melanospheres. (Figure 2d).

BAK-P cells were sorted by MACS into CD133(+) and CD133(−) subpopulations using anti-CD133-PE, and then exposed to increasing doses of targeted kinase inhibitors dabrafenib (a BRAF inhibitor) or trametinib (GSK1120212; a MEK inhibitor) for 72 h. XTT cell viability assays to assess drug sensitivity revealed that CD133(−) BAK-P cells were more sensitive to dabrafenib or trametinib compared to the CD133(+) BAK-P subpopulation (Figure 2e, left column). BAK-R cells were next compared to BAK-P cells with regard to their sensitivity to the same MAPK inhibitors. BAK-R cells, which express higher levels of CD133, were significantly much more resistant compared to BAK-P cells, comprised primarily of CD133(−) cells (Figure 2e, right column). These results together indicate that the CD133(+) cells represent cancer stem cells or MIC, exhibiting tumor initiation, increased expression of cancer stem cell and EMT markers, melanosphere formation, and chemoresistance to MAPK inhibitors. 

### 2.3. CRC BAK-R Melanoma Cells Exhibit Increased Invasiveness and Metastasis Compared to Parental Cells, Which Is Reversed by CD133 siRNA Knockdown

**Figure 3:**
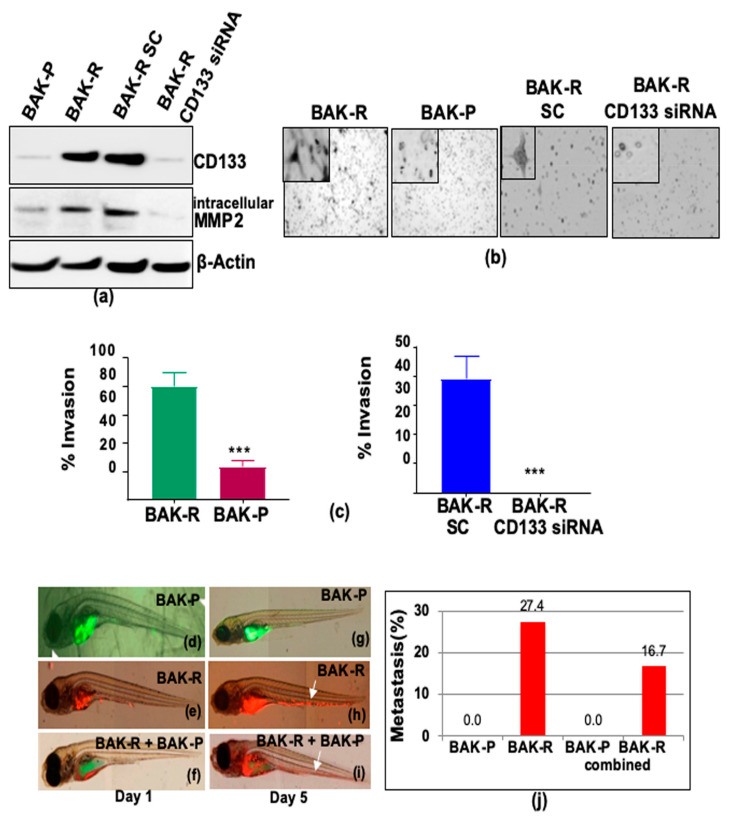
BAK-R cells exhibit increased invasion and metastasis in transwell invasion and zebrafish assays, compared to BAK-P (**b**,**c** left panels), while CD133 siRNA knockdown reduces CD133 and MMP2 expression (**a**) and inhibits invasion (**b**,**c** right panels) *** represent *p* < 0.001. (**c**) Transwell cell invasion assays were performed for 48 h. Cells that have invaded to the bottom of the transwell were stained with toluidine blue for imaging. Percentage of cell invasion was quantified and presented as a bar graph. Zebrafish were injected with the BAK-P (**d**) and BAK-R cells alone (**e**) or in combination (**f**), and imaged at day 1 (left panels) and day 5 (right panels). Quantification revealed that whereas parental cells showed no metastasis (**g**), reprogrammed CD133(+) BAK-R cells grafted alone (**h**), or in combination with parental BAK-P cells (**i**), exhibited significant metastasis to the tail (**j**).

The role of CD133 in invasion and metastasis was next assessed using the enriched population of conditionally reprogrammed patient-derived CD133(+) melanoma cells BAK-R. Compared to BAK-P cells, sustained upregulated CD133 expression in the BAK-R cell line was confirmed by immunofluorescence staining (Figure 1d) and immunoblot analysis (Figure 3a). Transwell invasion assays further showed that BAK-R cells are significantly more invasive than BAK-P cells (Figure 3b, left two panels; Figure 3c, left panel; *p* < 0.001). To further elucidate the role of CD133 in invasion, CD133 transcripts were knocked down in BAK-R cells using CD133-specific pooled siRNA, and compared to scrambled siRNA controls. Knockdown of CD133 expression in BAK-R cells by transfection with CD133 siRNA was confirmed by immunoblot analysis (Figure 3a). CD133-depleted cells exhibit diminished levels of intracellular MMP2 (Figure 3a). Transwell invasion assays, followed by quantification by Image J (Figure 3b right two panels, Figure 3c right panel), revealed a significant decrease in invasiveness of BAK-R CD133-siRNA knockdown cells compared to BAK-R-scrambled control cells (*p* < 0.001), indicating that CD133 plays an important role in invasiveness of BAK-R melanoma cells. 

The zebrafish model was next used to determine the metastatic potential of the different melanoma cell lines. BAK-R and BAK-P cells were transiently labeled with Vybrant DiI cell-labeling solution or CellTracker^TM^ Green 5-chloromethylfluorescein diacetate (CMFDA) dye, respectively, to distinguish the two cell types. Fluorescent-tagged BAK-P or BAK-R cells were then injected into zebrafish separately, or mixed 1:1 and injected together into the yolk sac of zebrafish to assess % metastasis to the tail (Materials and Methods). Zebrafish were injected with BAK-P (Figure 3d,g) or BAK-R cells (Figure 3e,h) alone, or in combination (Figure 3f,i), imaged under a fluorescent microscope, and scored for metastasis by migration of labeled cells to the tail (Figure 3j). BAK-P cells showed no metastases when grafted alone (Figure 3g), or in combination with BAK-R cells (Figure 3i). In contrast, BAK-R cells grafted alone (Figure 3h) or in combination with BAK-P cells (Figure 3i) exhibited significantly greater metastases to the tail (Figure 3j; *p* < 0.001; chi-square with Yates correction).

### 2.4. *CRISPR*-*Cas9* Knockdown of CD133 Significantly Reduces Invasion and Metastasis in BAK-R Cells

We further addressed the question as to whether elevated levels of CD133 in BAK-R play an essential role in the invasive and metastatic phenotype characteristic of tumor progression. We targeted three *CD133* coding region sites in exon 1 using CRISPR-Cas9 expressed in a lentiviral vector pLenti-U6-sgRNA-SFFV-Cas9-2A-Puro (Addgene; Supplementary Figure S1a (left); the vector expressed four different single guide RNAs (sgRNAs; Sc, T1, T2, and T3) to target each of the exon 1 sites, as described in Materials and Methods). Supplementary Figure S1a (right) shows the three regions of CD133 exon 1 that are targets of the T1–T3 sgRNAs cloned into the vector; a scrambled sgRNA was used as control. Single cell clones of BAK-R were isolated and selected with puromycin after transduction with lentivirus expressing Cas9 along with sgRNA for each of the 3 targets (T1, T2, and T3) along with control Sc sgRNA. However, since isolated clones of CD133+ cells lost CD133 expression over time, likely due to the number of population doublings required to establish a clonal population, we could not ascribe the loss of CD133 expression to specific disruption of the *CD133* gene. The experiment was thus modified to isolate pools of knockdown cells for each of the sgRNAs. These pooled clones were sequenced by NGS to determine the ­­­sequences at each sgRNA target site containing substitutions and indels (Supplementary Figure S1b). 

**Figure 4:**
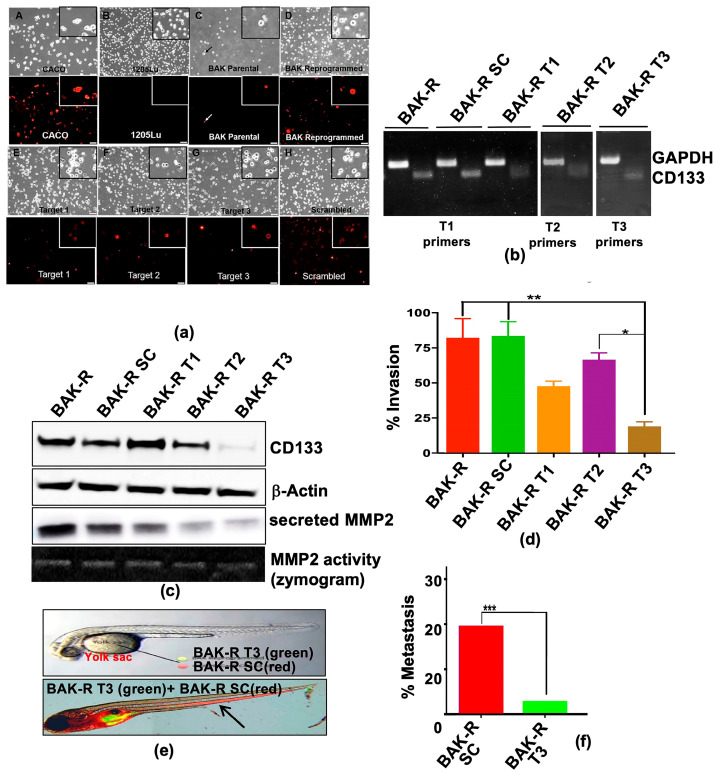
(**a**) CD133-depleted partial CRISPR-Cas9 BAK-R T3 cells show decreased CD133 expression and are less invasive and metastatic than BAK-R-SC controls. CD133 positivity was determined by immunofluorescence staining in BAK-P, BAK-R, partial CRISPR-Cas9 knockdowns using three target sgRNAs and a scrambled sgRNA control. CD133^+^ Caco-2 colon cancer cells and 1205Lu cells served as positive and negative controls, respectively. Images were taken at 10× magnification. RT-PCR (**b**) and immunoblot analysis (**c**) show depletion of CD133 RNA and protein in BAK-R T3 compared to BAK-R SC or BAK-R cells. (**d**) Transwell cell invasion assays showed decreased cell invasion in CD133-depleted BAK-R T3 cells, compared to BAK-R, control SC sgRNA, or T1 and T2 cells. (**e**) Injection of zebrafish with a 1:1 mixture of BAK-R SC (red) and BAK-R CD133 knockdown T3 cells (green) and representative images after 5 days; (**f**) quantification of % metastasis to the zebrafish tail. *, **, *** represent *p* < 0.05 and *p* < 0.01, and *p* < 0.001, respectively.

An initial screen for CD133-knockdown cells was performed by immunofluorescence analysis using anti-CD133/epitope 2 coupled to PE (Miltenyi Biotech, Bergisch Gladbach, Germany). Fluorescent staining of live cells harboring small CD133 insertions/deletions derived from scrambled, T1, T2, or T3 sgRNA target sequences reveal that T3 displays the least CD133(+) cells (Figure 4a), possibly the result of the large percentage of frameshift mutations resulting from targeting T3 (Supplementary Figure S1b). RT-PCR for the CD133 RNA was further performed using primer pairs that amplified a PCR product small enough from each target of exon 1 to detect the small indels in each pooled population. RT-PCR using primers flanking the sgRNA targets show reduced CD133 expression, and multiple-sized bands indicating different sized deletions in the gene and corresponding RNA, whereas amplification of the scrambled control reveals a single band (Figure 4b). The levels of CD133 protein expression were then examined by immunoblot analysis. CD133 protein levels were barely detectable in BAK-R-T3 cells (Figure 4c), perhaps reflecting a higher percentage of frameshift mutations compared to BAK-R-T1 and BAK-R-T2 (Supplementary Figure S1b). In contrast, BAK-R-T2 cells express intermediate levels of CD133 protein, and BAK-R-T1 and BAK-R-Sc express high levels of CD133, similar to those of BAK-R cells. 

Having achieved near complete knockdown of CD133 protein in BAK-R-T3 cells, transwell invasion assays were next performed to examine the invasiveness and metastatic potential of the CRISPR-CD133-depleted cells compared to BAK-R-SC control cells. Whereas BAK-R and BAK-R-SC controls were the most invasive, invasion of BAK-R-T3 was significantly attenuated (*p* < 0.001; Figure 4d). Zebrafish assays further revealed significantly lower metastasis in BAK-R-T3 cells compared to scrambled controls (Figure 4e,f; *p* < 0.001, chi square), indicating an essential role of CD133 in metastasis of BAK-R cells. 

Matrix metalloproteinases MMP2 and MMP9 are zinc-dependent enzymes that cleave components of the ECM. MMP genes encode gelatinase A, type IV collagenase, which cleave Type IV collagen, the main component of basement membrane. Previous studies have implicated upregulation of MMP expression in invasion of surrounding tissues. We next examined whether increased invasiveness of CD133(+) cells is mediated by MMPs secreted by these cells. Immunoblot analysis with antibodies to MMP2 and MMP9 revealed that enhanced invasion of CD133(+) enriched BAK-R cells may, at least in part, be due to upregulation of MMP2 and MMP9 expression (Figure 2a). Furthermore, intracellular or secreted MMP-2 levels are markedly diminished in CD133-depleted BAK-R-CD133 siRNA (Figure 3a) or BAK-R-T3 cells (Figure 4c), compared to SC controls or BAK-R, suggesting that the effects of CD133 on invasion may be mediated by MMP2. 

### 2.5. CRISPR-Cas9 Knockdown of CD133 Expression in BAK-P Melanoma Cells Attenuates Cell Invasion and Decreases Levels of MMP9 and MMP2 

We investigated the role of CD133 in the invasiveness of MICs, and whether this is mediated by MMPs secreted by these cells. CD133 was knocked out by lentiviral delivery of CRISPR-Cas9 associated sgRNAs targeting any of three loci (T1, T2, and T3) in exon 1 of the PROM1 gene in BAK-P cells. Frameshift mutation analysis from NGS revealed that while most mutations generated in T1 and T2 cells were in-frame mutations, 79% of mutations in T3 cells were frameshift mutations (Supplementary Figure S2a). In addition, while allelic mutations at CRISPR target sites in T1 and T2 cells were only deletions, T3 cells exhibited both insertions and deletions (Supplementary Figure S2b). 

**Figure 5:**
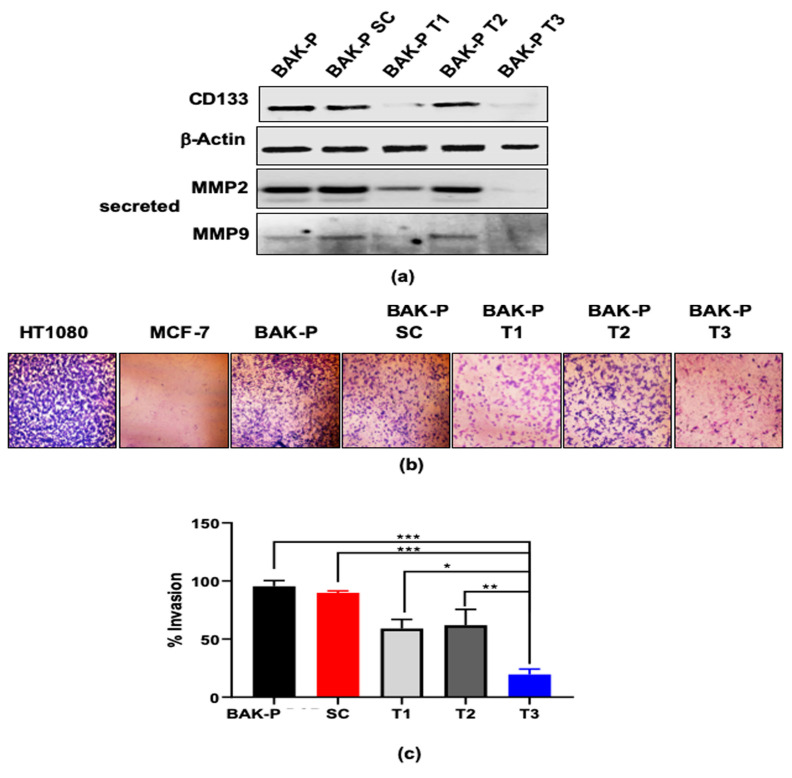
(**a**) Immunoblot analysis showing protein levels of CD133, as well as secreted MMP2 and MMP9, in different CRISPR-Cas9 melanoma cell lines and BAKP; β-Actin verified equal protein loading. Immunoblot analysis shows decreased levels of CD133 and secreted MMPs in CRISPR Cas-9 CD133 knockdown melanoma pooled clone T1 and T3, compared to BAKP and scrambled control cells. (**b**) Representative images of crystal violet stained invading cells after 48 hours in transwell invasion chambers; HT1080 and MCF-7 served as positive and negative controls, respectively. (**c**) Percent invasion of CRISPR Cas-9 CD133 knockdown cell lines (T1, T2, T3) compared to BAKP or scrambled (SC) control cells; *, **, *** represent *p* < 0.05, *p* < 0.01, or *p* < 0.001 respectively compared to T3 clone.

Partial CD133 knockdown was confirmed in pooled clones of BAK-P-T1, while BAK-P-T3 cells showed almost no detectable CD133 expression in immunoblots (Figure 5a), similar to the results with BAK-R cells. Boyden chamber invasion assays were then performed to assess the effect of CD133 knockdown on the invasive potential of these melanoma cells. Cell invasion was attenuated in the BAK-P-T1, and T3 cells, compared to BAK-P or scrambled sgRNA control cells (Figure 5b). Quantification of invasiveness corresponds to levels of immunodetectable CD133; e.g., CD133-depleted BAK-P-T3 cells were the least invasive (Figure 5c). Immunoblot analysis with antibodies to MMP9 and MMP2 further revealed that CRISPR-Cas9 knockdown of CD133 in BAK-P-T3 cells results in significantly diminished levels of secreted MMP2 and MMP9 (Figure 5a). Thus, there may be a positive correlation between CD133 expression and secreted MMP2/MMP9. Invasiveness of CD133-expressing MICs may be mediated by upregulation and activation of MMP2/MMP9.

### 2.6. Doxycycline-Inducible Expression of CD133 Levels in BAK-P Melanoma Cells Increases Cell Invasion and Levels of Secreted MMP9 and MMP2

**Figure 6:**
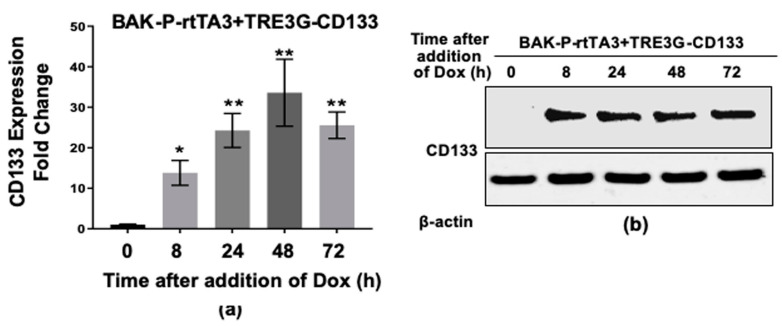

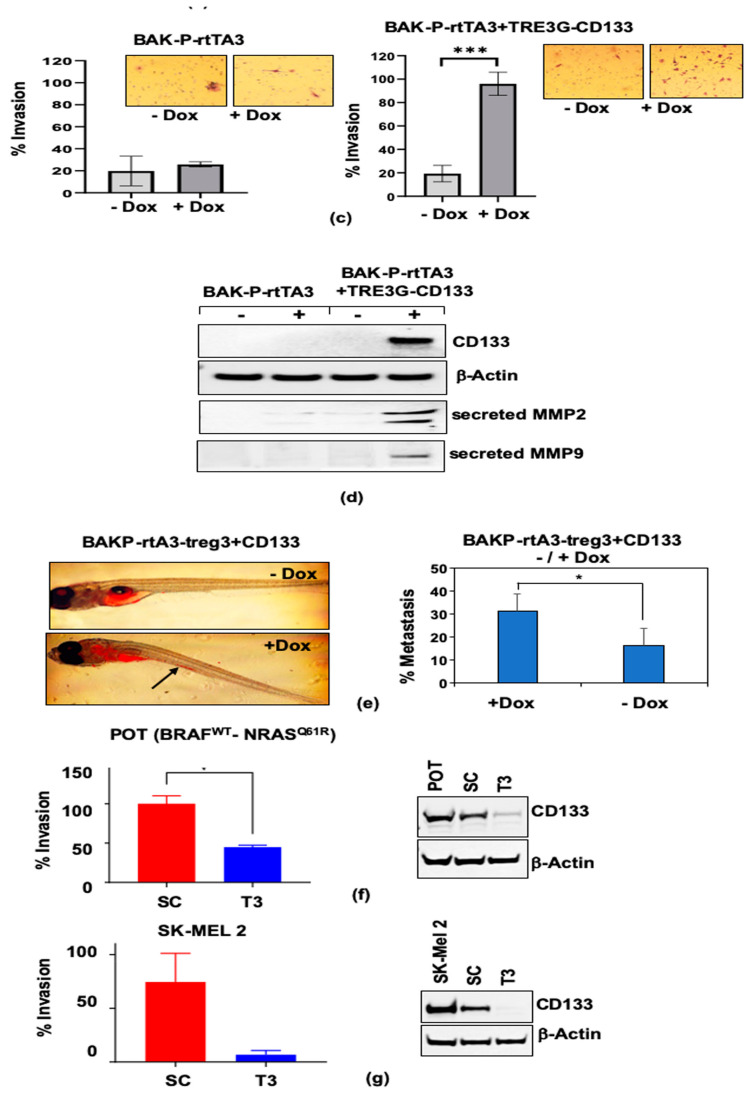
Dox-inducible CD133 expression in BAK-P cells as verified by qPCR (**a**) and immunoblot analysis (**b**). (**c**) Dox-inducible CD133 expression significantly increases cell invasion in transwell assays. (**d**) Immunoblot analysis with antibodies to CD133, MMP9, MMP2, or β-Actin for loading controls, show increased levels of CD133 as well as MP2 and MMP9 secreted by Dox-induced cells. (**e**) Zebrafish assays reveal enhanced metastasis in Dox-induced cells. (**f**,**g** left panels) Invasion assay results with other melanoma cell lines POT (**f**) and SK-MEL2 (**g**) CD133 CRISPR-cas9 SC versus T3 lines are consistent with results with BAK-P cells, showing that knockdown of CD133 (**f**,**g** right panels) results in decreased cell invasion. *, **, *** represent *p* < 0.05 and *p* < 0.01, and *p* < 0.001, respectively.

To further confirm the pathway between CD133, MMP, and invasion, BAK-P cells, which express low levels of CD133, were co-transduced with a Tet activator (rtTA3) and a Tet-on vector expressing CD133 (TRE3G-CD133), and selected with blasticidin and gentamycin. Stable pooled clones of BAK-P-Tet-CD133 were then cultured in the absence or presence of doxycycline (Dox) for increasing amounts of time. Dox induced marked expression of CD133 RNA (Figure 6a) and protein (Figure 6b) by 8 h in a time-dependent fashion. Transwell invasion assays revealed an increase in invasiveness of BAK-P-Tet-CD133, but not BAK-P-rtTA3, only in the presence of Dox (Figure 6c); quantification revealed the difference to be significant (*p* < 0.001), indicating that ectopic expression of CD133 is responsible for the increased invasion of BAK-P cells. Immunoblot analysis further shows that this corresponds to increased levels of MMP2 and MMP9 secreted by the cells (Figure 6d).

Zebrafish assays were next performed to assess the metastatic activity of the cells following induction of CD133 expression. BAK-P-Tet-CD133 cells were transiently labeled with Vybrant DiI Cell-Labeling Solution (ThermoFisher, V22885, Waltham, MA, USA) with or without Dox induction of CD133, and injected into each fish to compare rates of metastasis (Materials and Methods). While BAK-P cells showed minimal metastases in the absence of Dox, metastasis increased significantly after Dox-inducible CD133 expression (Figure 6e; *p* < 0.001; chi-square with Yates correction).

To validate the suppressive effects of CD133 knockdown by CRISPR-Cas9 on invasiveness of melanoma cells, we compared the consequences of CD133 knockdown in other melanoma cell lines. We derived SC controls and compared them to CRISPR-Cas9 T3 knockdowns in these cell lines. As with BAK-R and BAK-P cells, POT (Figure 6f) and SK-Mel 2 (Figure 6g) melanoma cells showed marked reduction in immunodetectable CD133 in T3 cells compared to scrambled controls. Consequently, invasion was also significantly attenuated in the T3 cells compared to SC controls, lending strong support to an essential role of CD133 in invasion in multiple melanoma lines, including BAK-P, BAK-R, SK-Mel2, and POT cells (Figure 6f,g left panels). 

## 3. Discussion

In our previous study, we showed that CD133 levels were correlated with recurrent disease, poor clinical outcomes, and decreased overall survival; CD133(+) cells formed tumors in nude mouse xenografts, whereas CD133(−) cells did not [34]. We have also shown that expression of CD133 is associated with the upregulation of ABCBG2 and drug resistance [33]. In the current study, we determined whether CD133 itself was contributing to tumorigenesis and progression, or whether other co-expressed genes were responsible. When mixed populations of CD133(+) and CD133(−) cells were xenografted in nude mice, CD133(+) MIC cells were found to contribute to tumor formation.

Further studies in cultured cells were difficult, since the expression of CD133 was reduced over time, even after plating MACS-purified CD133(+) cells. We therefore utilized CRC in order to stabilize the CD133(+) phenotype for additional studies. The CRC-generated BAK-R cells maintained high levels of CD133 expression, along with stem cell markers, including Oct4 and Nanog, and grew entirely in large melanospheres in detached cultures. Stem cell marker expression and melanosphere growth were also characteristic of freshly MACS-sorted CD133+ cells. This is consistent with previous studies that have shown that the CRC protocol does not alter the cells previously tested in terms of copy-number variations, or mutations [65,66]. Further, in common with MACS-isolated CD133(+) cells, BAK-R showed increased resistance to MAPK inhibitors. Compared to BAK-P cells, BAK-R showed an increased ability to invade, as well as increased metastasis in zebrafish assays. The contribution of CD133 to the invasiveness and metastasis of melanoma cells was validated by CD133 depletion by CD133 siRNA, as well by CRISPR-Cas9 knockdown of CD133 in BAK-R cells, both of which inhibited invasion, while metastasis was also inhibited using CD133 CRISPR-Cas9 knockdown. A potential mechanism for increased invasion was demonstrated by upregulated levels of intracellular or secreted active metalloproteinases MMP2 and MMP9 in BAK-R cells, which was suppressed in BAK-R-CD133 siRNA as well as BAK-R CRISPR-Cas9 T3 knockdowns. 

One advantage of CRISPR-Cas9 over siRNA is the stability of the CRISPR-Cas9 lines, which exhibited reduced CD133 expression long-term. This allowed us to perform longer-term metastatic assays, which sometimes require up to a week in zebrafish. A second advantage of using CRISPR-Cas9 knockdown approach to supplement our siRNA studies is that we were able to create three CRISPR-Cas9 knockdown lines (T1, T2, and T3) that reproducibly express different levels of CD133, with T3 cells in all four lines expressing the lowest levels of CD133. This may be due to the large number of frameshift indels in T3 compared to T1 and T2 (Supplementary Figure S1). This allowed us to correlate different CD133 levels with biological activities of each of the CRISPR knockdowns. Variations in CD133 expression levels are important, since we have previously shown that patient ‘days to death’ are significantly inversely correlated with CD133 levels [33], and percentages of cells expressing CD133 are correlated with other negative clinical outcomes [34]. In contrast, siRNA levels cannot be used to reproducibly express varying levels of CD133. Third, as we have shown, any further studies that might utilize typical single clone CRISPR selection to select for CD133 knockout will unwittingly select for epigenetic changes that have nothing to do with the knockout itself. Thus, comparisons between scrambled, T1, T2, and T3 clones would be meaningless. Fourth, when we cultured pooled clones of scrambled—T1, T2, and T3 cells under stem conditions—all scrambled or non-transduced control pooled clones exhibited high levels of CD133, as in the case of BAK-R. Even under these conditions, we were able to sufficiently and stably knockdown CD133 levels with CRISPR-Cas9 to observe changes in invasion and metastasis. Finally, similar to miRNAs, the tolerance for siRNA mismatches has been a challenge when targeting genes for suppression in research and clinical applications [67,68], whereas this issue is not as inherent in DNA-editing systems including CRISPR-Cas9. We therefore employed a combination of approaches by supplementing the siRNA and CRISPR-Cas9 knockdown studies with Dox-inducible CD133 expression. Using these approaches together more rigorously demonstrated an essential role for CD133 in invasion, metastasis, and tumor progression.

In BAK-P cells, we were also able to demonstrate decreased invasion due to CD133 depletion in CRISPR-Cas9 T3 knockdowns. As with BAK-R, BAK-P secreted matrix metalloproteinase MMP2 and MMP9, which was markedly suppressed by CD133 CRISPR-Cas-9 knockdown. Dox-inducible CD133 expression in BAK-P cells upregulated active MMP2 and MMP-9 secretion, and significantly enhanced invasion, confirming the role of a CD133-MMP axis in tumor progression and explaining the importance of our original finding of a significant correlation between CD133, Breslow thickness, recurrent disease, poor clinical outcomes, and short overall survival [34]. These results were further validated when we derived CD133 knockdown cells in two additional melanoma cell lines, and demonstrated reduced invasion in these CD133-depleted cells.

CD133 is associated with several specific pathways, including the MAPK and TGFβ pathways. We have performed microarray analysis of CD133(+) and CD133(−) cells separated by MACS, as well as BAK-R cells (>80% CD133+) compared to BAK-P cells, which contains approximately 5% CD133+ cells. In both cases, the TGFβ and MAPK-associated genes were found to be differentially expressed in CD133(+) cells (data to be published elsewhere). CD133 appears to be controlled upstream by CD9. 

The pathway from CD133 to MMPs, invasion and metastasis remains to be elucidated. In order for cells to form metastases, they must be able to first invade the basement membrane, presumably with the aid of MMPs. Overexpression of matrix metalloproteinases, especially MMP2 and MMP9 appear to be particularly important in melanoma invasiveness [37,38,39]. A key role played by MMP9 was demonstrated in studies that showed that this protease promotes melanoma invasiveness by degrading components of the extracellular matrix [40,41,42,43]. MMP9 expression is regulated by several pathways and epigenetic alterations [44,45,46]; overexpression is due to the aberrant activation of the MAPK and AKT/mTOR signaling pathways almost always found in melanoma [47,48]. HIF-1α and HIF-2α have also been shown to activate the CD133 promoter through ETS proteins [69]. MMP9 expression is also regulated by several miRNAs [49,50].

CD133 expression has been shown to be inversely correlated with tissue inhibitors of metalloproteinases (TIMP-2), providing a link between CD133 expression and MMPs [70]. In the current study, MMP2 and MMP9 themselves were upregulated by CD133, although MMP2 and/or MMP9 secretion may be coregulated with TIMP-2 decrease; they are not mutually exclusive. CD133 has also been associated with vasculogenic mimicry, distant metastasis and poorer prognosis in salivary adenoid cystic carcinoma (ACC) [71]. Consistent with the current study, CD133(+) MIC showed increased migration and invasion compared to CD133(−) ACC cells. Similarly, co-expression of CD133, MMP2, and MMP9 was also observed in triple-negative breast cancer cells [31]. Metastasis of liver cancer is closely linked to tumor microenvironment, in which chemokines and their receptors play an important role [71]. CXCR3, the receptor of chemokine CXCL9, is frequently upregulated in HCC and correlates with tumor size, tumor differentiation, portal invasion, and metastasis. The CXCR3-A isoform bound by CXCL9 upregulates MMP2 and MMP9 expression, and promotes invasion and metastasis of CD133(+) liver cancer cells.

Future studies will attempt to connect the mechanism of MMP upregulation with CD133 expression. Additional metastasis-specific gene pathways will be examined using our BAK-P -Tet-CD133 cells, including those regulated by TGFβ, which we have found to be important after microarray analysis comparing BAK-R vs. BAK-P. We have also previously shown the importance of the TGFβ/ID pathway in melanomagenesis [72,73]. Given that MMP9 is also regulated by miRs [49,50], the differential miR expression in CD133(+) versus CD133(−) melanoma cells or after Dox-induced CD133 expression will be further examined.

## 4. Materials and Methods 

### 4.1. Cells

Melanoma initiating cells (MIC) were isolated from human melanoma cell lines harboring different kinase mutations and included: BAK-P (BRAF^WT^, NRAS^Q61K^), POT (BRAF^W^, NRAS^Q61R^). These cells were established from fresh lymph node metastases from patients with poor clinical outcomes including short overall survival (<10 months). Cell suspensions were prepared by mechanical dissociation by repeated mincing with scalpels and scissors in Iscove’s medium containing 10% FBS and antibiotics (1% penicillin-streptomycin), and analyzed for the melanoma-specific antigens MART1 and S100 by flow cytometry using monospecific antibodies. Cells were maintained in Iscove’s modified Dulbecco’s medium (IMDM) supplemented with 10% FBS and 1% penicillin-streptomycin and maintained in a 37 °C 5% CO_2_ humidified incubator. Cells were grown to 70–80% confluency and then passaged 1:4 (two population doublings). To compare cells at similar population doublings, large numbers of cells of identical passage numbers were routinely frozen, and after several passages, the presence of BRAF^V600^ or NRAS^Q61^ in each of the parental cell lines was verified by direct Sanger DNA sequencing of PCR products, as described previously [33].

### 4.2. Magnetic Sorting and Pre- and Post-Staining for CD133-Positivity

Early passages (<20) of parental cell culture suspensions of either non-transduced, GFP- or DsRed-expressing melanoma cells were incubated with anti-CD133 (epitope 2) conjugated to PE (clone REA816; Miltenyi Biotec, Waltham, MA, USA) and visualized by fluorescence microscopy to determine CD133 positivity prior to magnetic-activated cell sorting (MACS^®^). Total and viable cell counts were performed by trypan blue staining and cell counting with an Eve^TM^ automatic cell counter (NanoEnTek, NanoEnTek, Inc. (USA), Waltham, MA, USA, a subsidiary of NanoEnTek, Inc., South Korea). CD133(+) and CD133(−) subpopulations were then separated using paramagnetic nanobeads conjugated to anti-CD133 and a MACS^®^ Column (Miltenyi Biotech) according to manufacturer’s specifications. To increase the purity of sorted populations, the CD133(+) cells were further purified over a second MACS^®^ column. For non-transduced cells, sorted CD133(+) and CD133(−) subpopulations were subjected to post-MACS staining with anti-CD133-PE (epitope 2; Miltenyi Biotec) and fluorescence microscopy to manually quantify CD133 positivity in sorted populations. 

To assess CD133 positivity in sorted cells expressing fluorescent proteins, DsRed-expressing CD133(+) cells were incubated with anti-CD133 (Miltenyi Biotec, Bergisch Gladbach, Germany), followed by Alexa 488 conjugated to goat anti-mouse IgG, while untransfected or GFP-expressing CD133(−) cells were stained with CD133-PE-conjugated antibody, followed by manual counting of transduced fluorescently labeled cells under an Olympus fluorescent microscope, or automated counting using Countess-FL (Invitrogen, Carlsbad, CA, USA). Additionally, photomicrographs were obtained and percentages of fluorescent cells were assessed using Image J (https://imagej.nih.gov/ij/download.html). “Caco-2 (ATCC HTB-37), a continuous colon cancer cell line of heterogenous human epithelial colorectal adenocarcinoma cells expressing CD133 in >80% of the cells, was used as a CD133(+) positive control [74], while CD133(−) 1205 Lu cells, a melanoma cell line, served as negative control. 1205-Lu cells is a variant cell line derived from a primary melanoma cell line (WM 793) selected for metastatic phenotype in athymic mice [75].” 

Flow cytometry was also performed to confirm the sorted populations as CD133(+) or CD133(−) using mouse mAb monospecific for epitope 2 (CD133/2; Miltenyi Biotech). 

### 4.3. Formation of Melanospheres 

To confirm formation of melanospheres, a cancer stem cell feature, cells were grown in DMEM/F-12 (1:1) supplemented with EGF and FGF (Invitrogen) in ultra-low attachment plates (Corning, NY, USA).

### 4.4. Conditional Reprogramming of CD133(+) Cells

To capture a subpopulation of CD133(+) MIC cells with sustained CD133 overexpression, magnetically sorted CD133(+) cells were conditionally reprogrammed using the ‘Georgetown Method’. CD133(+) 3D melanosphere cultures were maintained in 60 mm ultra-low attachment plates with DMEM-F12 stem cell media, supplemented with 10 ng/mL of EGF and FGF, heparin (2.5 μg/mL), B-27, Y-27632 (5 μM), and penicillin/streptomycin. CD133-sorted cell populations were incubated for a month in 3D conditions in hypoxic (2% O_2_) conditions at 37 °C and 5% CO_2_. After a month in 3D melanosphere conditions, cells were transferred to attached 2D conditions in 100 mm plates with DMEM-F12/FY media. DMEM-F12/FY consist of 1 FY: 2 F12 media; FY media was prepared with DMEM with 10% FBS, 1% Glutamine, F12 nutrient mix, hydrocortisone/EGF mix, insulin (5 mg/mL), fungizone (250 μg/mL), gentamicin (10 mg/mL), cholera toxin (0.1 μM), and Y-27,632 (10 μM). F12 media consist of F12 nutrient mix, 1% penicillin/streptomycin, 7% FBS, TPA/PMA (50 ng/mL), 3-isobutyl-1-methylxanthine (IBMX; 0.1 mM), Dibutyryl cyclic adenosine monophosphate (*dBcAMP*; 0.1 mM), cholera toxin (2.5 nM), and endothelin 1 (10nM). These 2D cultures were then incubated at 37 °C, 5% CO_2_, and allowed to recover and expand under attached conditions for a month before CD133 immunofluorescence staining.

### 4.5. siRNA Knockdown

Knockdown experiments were performed according to standard protocols using small interfering RNAs (siRNAs) specific for CD133 or scrambled siRNA controls (Life Technologies, Carlsbad, CA, USA). The sequences used were as follows:


**Cat #**

**Sequence**
Flexitube siRNA PROM1 1SI00083741CACGTTATAGTCCATGGTCCAFlexitube siRNA PROM1 2SI00083748CAGGTAAGAACCCGGATCAAAFlexitube siRNA PROM1 3SI00083755ACCTTTGAGTTTGGTCCCTAAFlexitube siRNA PROM1 4 SI03098263CTGGCTAAGTACTATCGTCGA

Successful knockdown was verified by immunoblot and qRT-PCR analyses.

### 4.6. Transwell Migration/Invasion Assay

5 × 10^5^ cells per well were seeded in Matrigel Invasion chambers (Corning), according to manufacturer’s specifications. Cells were seeded in the upper chamber of experimental wells that have Matrigel-coated inserts as well as in control uncoated wells. Invasive cells secrete proteases that degrade the Matrigel matrix and enable invasion through the membrane pores. FBS in IMDM media was added to the lower chamber as a chemo-attractant, and cells were allowed to migrate or invade for 48 h. Noninvasive cells were then scrubbed from the top of the insert with sterile cotton swabs; metastatic cells that have migrated to the bottom of the membrane were fixed with methanol and stained with toluidine blue. Quantification was performed by direct counting as well as by Image J analysis. Cell invasion (%) was computed as cells that migrated in experimental Matrigel-coated wells over those that migrated in uncoated control wells ×100.

### 4.7. Quantitative Reverse-Transcription PCR (qRT-PCR)

Total RNA purified from cell pellets with Trizol Reagent (Gibco BRL, Grand Island, NY, USA) were subjected to qRT-PCR by standard protocols using two-step reverse transcription-PCR (Invitrogen), 0.75 μg of RNA and specific primers:

CD133 forward-5ʹ-CCC GGG GCT GCT GTT TAT A
CD133 reverse-5ʹ-ATC ACC AAC AGG GAG ATT G
GAPDH forward 5ʹ-GAA GGT GAA GGT CGG AGT C
GAPDH reverse 5ʹ-C GAA GAT GGT GAT GGG ATT TC

### 4.8. Mouse Xenografting 

All animal experiments were performed in accordance with the guidelines and approval of Georgetown University Institutional Animal Use and Care Committee (protocol/project identification number 2016-1218). This committee is responsible for the dissemination of information related to approved methods of animal care to individuals who use animals in research and teaching at Georgetown University. Athymic *NCr*-*nu/nu* 6-week old male mice (Harlan Laboratories, Indianapolis, Indiana) were acclimated to the Division of Comparative Medicine at Georgetown University a week prior to xenografting. 500,000 CD133(−) MACS-sorted DsRed BAKP cells were mixed with an equal number of CD133(+) GFP cells resuspended in Matrigel matrix (Corning), and injected subcutaneously into the hind flanks of nude mice using a 20-gauge syringe. Mice were monitored for tumor growth using the formula: tumor volume = 1/2 length x width^2^, where width is the smaller dimension of the tumor as described previously [76,77]. Cells were also monitored for green or red fluorescence using the Maestro Imager (Cambridge Research & Instrumentation, Inc. (*CRi*, Hopkinton, MA, USA).

### 4.9. Zebrafish Injection and Imaging

Transparent zebrafish eggs were dechorionated using 200 μg/mL pronase. After 24 hours of development, zebrafish embryos were arranged in agar injection plates and anesthetized with 2× tricaine, prepared from a 4 mg/mL stock and diluted in fish water. Cells were transiently labeled with Vybrant DiI Cell-Labeling Solution (ThermoFisher, V22885, Waltham, MA, USA) or Molecular Probes CellTracker^TM^ Green CMFDA (5-chloromethylfluorescein diacetate) Dye (Life Technologies, C2925, Carlsbad, CA, USA) to distinguish the different cell types (parental vs. reprogrammed vs. CRISPR-Cas9 CD133 knockdown). A 1:1 ratio of 100–200 fluorescently cells labeled with either Cell Tracker Green CMFDA Dye or vibrant DiI red cell-labeling solution, were injected into the yolk sac of the developing embryo using a glass microinjection needle. Around 100 to 200 fluorescent cells were injected into the embryonic yolk sacks of each of 30 anesthetized zebrafish embryos. After injection and recovery, each fish was transferred to a well in a 96-well plate. Fluorescent images of zebrafish were taken to assess overall fish health, injection success, and initial location of cells. Zebrafish were then imaged again under a fluorescence microscope at indicated times to assess cell metastasis characterized by cell migration to the tail.

### 4.10. CRISPR-Cas9 Knockdown of CD133

Three different sgRNA sequences specific for the first exon of CD133 in lentiviral vector pLenti-U6-sgRNA-SFFV-Cas9-2A-Puro (Addgene) were packaged and transduced into melanoma cells. Lentiviral transduction and CRISPR-Cas9 knockdown: HEK 293 cells were transfected with pLenti-U6-sgRNA-SFFV-Cas9-2A-Puro plasmid (4 µg) containing individual signal-guide sequences (sgRNA) beginning at either 8, 69, or 205 bp downstream of the beginning of the CD133 coding sequence, all within the first exon coding sequence of CD133, as shown below:

sgRNA1: CAACAGGGAGCCGAGTACGA (complement of Target 1 underlined below)
sgRNA2: TTCATCCACAGATGCTCCTA (complement of Target 2 underlined below)
sgRNA3: TTACCTTCTGGGAAATCACGC (complement of Target 3 underlined below)

CD133 coding sequence of first exon with targets T1, T2, and T3 underlined:

ATG GCC CTC GTA CTC GGC TCC CTG TTG CTG CTG GGG CTG TGC GGG AAC
                 **T1**
TCC TTT TCA GGA GGG CAG CCT TCA TCC ACA GAT GCT CCT AAG GCT TGG
                     **T2**
AAT TAT GAA TTG CCT GCA ACA AAT TAT GAG ACC CAA GAC TCC CAT AAA GCT GGA CCC ATT GGC ATT CTC TTT GAA CTA GTG CAT ATC TTT CTT ATG TGG TAC AGC CGC GTG ATT TCC CAG AAG GTA A
                **T3**

HEK 293 cells were transfected along with lentivirus packaging plasmids using Lipofectamine LTX (Thermo Fisher Scientific, Waltham, MA, USA). Lentivirus was harvested in media after 2 days and applied to melanoma cells. Transduced cells were selected with puromycin (4 µg/mL) for 5 days, and pooled and individual clones were derived and subjected to PCR analysis of genomic sequences. Alterations of the first exon was performed by PCR amplification followed by Sanger sequencing, as well as by NGS sequencing to assess allelic or frameshift mutations (%) at CRISPR target sites T1, T2, and T3. 

To directly observe CRISPR-Cas9 deletions in pooled cells, the following primers flanking human genomic Targets 1, 2, or 3 (T1, T2, T3) were amplified as small PCR products, where small NHEJ deletions could be visualized on 15% urea polyacrylamide gels.

Target 1-forward (F1a) TTCCCCAAGGCTTCCAGAAG
Target 1-reverse (R1a) GCCCTCCTGAAAAGGAGTTC
Target 2-forward (F2a) GAACTCCTTTTCAGGAGGGC
Target 2-reverse (R2a) GAGAATGCCAATGGGTCCAG
Target 3-forward (F3a) CTGGACCCATTGGCATTCTC
Target 3-reverse (R3a) CATTCT1TCCCTGCCATCAGC

### 4.11. Generation of Dox-Inducible Cells 

To generate the Dox-inducible lentivirus that can induce CD133 expression, psPAX2 and pMD2.G (5 µg each; Addgene, Cambridge, MA, USA) and pLenti-CMV-rtTA3 Blast (Addgene), with or without pLV-EGFP/Neo- TRE3G-CD133 (10 µg; VectorBuilder Inc., Chicago, IL, USA) were co-transfected into HEK293FT cells using Lipofectamine LTX (Thermo Fisher Scientific) according to the manufacturer protocols. Cells were provided with fresh medium after 16 h, and incubated for another 48 h to produce the lentivirus. Cell supernatant was then collected, centrifuged to concentrate the virus, and filter-sterilized with a 0.22 µm filter prior to use. For the virus transduction, cells were seeded into 6-well plates and viral supernatants were added to the media at MOI = 1. After 24 h, the virus-containing media was replaced with cell culture media, and transduced cells were then selected after 48 h with blasticidin (40 µg/mL) and gentamicin (1000 µg/mL) for 10 days.

### 4.12. Immunoblot Analysis

SDS-polyacrylamide gel electrophoresis (PAGE) and western transfer to nitrocellulose membranes were performed according to standard procedures. Ponceau staining of membranes verified equal loading and transfer of proteins. To assess protein levels of CD133, cancer stem cell markers Oct4, Nanog, EMT marker Vimentin, as well as MMP2 and MMP9 protein expression. Membranes were probed with antibodies specific for CD133 (Miltenyi Biotech, Bergisch Gladbach, Germany); Oct4, Nanog, Vimentin (Abcam, Cambridge, UK); MMP2, MMP9, β-Actin (Proteintech Group, Inc., Rosemont, IL, USA). β-Actin was used to verify equal protein loading. For secreted MMP2/MMP9, conditioned media was collected from the cells, concentrated by centrifugation at 14,000 g using 10 kDa Amicon^®^ filters (MilliporeSigma, Burlington, MA, USA), and then separated by SDS-PAGE, and western transfer. Immunoblots were then incubated with appropriate horseradish peroxidase-conjugated anti-mouse or rabbit IgG, followed by enhanced chemiluminescence with *Pierce* ECL Western Blotting *Substrate* (Thermo Fisher Scientific, Waltham, MA, USA), detection of immune complexes with a ChemiDoc™ Gel Imaging System (Bio-Rad Laboratories, Hercules, CA, USA), and densitometric analysis with Image J. 

### 4.13. Statistical Analysis

All assays were performed in triplicate. Curve fitting graphs were generated with Prism (GraphPad Software, San Diego, CA, USA). Error bars are reported as standard deviations of triplicates and *p*-values were calculated using a standardized *t*-test. *p*-values of <0.05 were considered statistically significant and represented with a single asterisk, while *p* < 0.01, *p* < 0.001, or *p* < 0.0001 are shown as 2, 3, or 4 asterisks respectively. The results are representative of three independent experiments with reproducible results.

## 5. Conclusions

CD133 induces xenograft tumors in mixed populations. Conditional reprogramming stabilizes the expression of CD133, which behave similarly to CD133(+) cells derived by MACS-sorting of tumor cells, as both express abundant CD133, form huge melanospheres, express stem cell markers, and are resistant to kinase inhibitors. BAK-R cells demonstrate CD133 siRNA-reversible MMP expression, invasion, as well as increased metastasis. Invasion, MMP expression, and metastasis of BAK-R and BAK-P are attenuated by CRISPR-Cas9 knockdown of CD133, as is invasion of two other patient-derived melanoma cell lines. Invasion and metastasis are increased in BAK-P cell by the Dox-inducible expression of CD133. These data strongly indicate an essential role for CD133 in MMP expression, invasion, and metastasis, and further support the use of CD133 as a target for therapeutic intervention. 

## Supplementary Materials

The following are available online at https://www.mdpi.com/2072-6694/11/10/1490/s1, Figure S1: (a) CRISPR Cas9 pLenti plasmid from ABM and Gene Targets, (b) Frameshift mutation analysis; Figure S2: (a) Frameshift mutation analysis from Next Generation Sequencing and (b) visualization of allelic mutations at CRISPR target sites; Figure S3: Scans of whole gel western blots showing molecular weight sizes of relevant proteins (indicated in arrows) for Figures 1f, 2a, 3a, 4c, 5a, 6b, 6d, 6f, and 6g; Figure S4: Densitometry analysis of western blots; Figure S5: RT-PCR gel images for Figure 4b.
