# Correction: Simbulan-Rosenthal et al. CRISPR-Cas9 Knockdown and Induced Expression of CD133 Reveal Essential Roles in Melanoma Invasion and Metastasis. *Cancers* 2019, *11*, 1490

**DOI:** 10.3390/cancers17213436

**Published:** 2025-10-27

**Authors:** Cynthia M. Simbulan-Rosenthal, Ryan Dougherty, Sahar Vakili, Alexandra M. Ferraro, Li-Wei Kuo, Ryyan Alobaidi, Leala Aljehane, Anirudh Gaur, Peter Sykora, Eric Glasgow, Seema Agarwal, Dean S. Rosenthal

**Affiliations:** 1 Department of Biochemistry and Molecular & Cellular Biology, Georgetown University School of Medicine, Washington, DC 20007, USA; simbulac@georgetown.edu (C.M.S.-R.); rdd40@georgetown.edu (R.D.); sv457@georgetown.edu (S.V.); amf315@georgetown.edu (A.M.F.); lk702@georgetown.edu (L.-W.K.); raa125@georgetown.edu (R.A.); la591@georgetown.edu (L.A.); ag854@georgetown.edu (A.G.); 2 Amelia Technologies, Rockville, MD 20850, USA; peters@ameliatechnologies.com; 3 Department of Oncology, Georgetown University School of Medicine, Washington, DC 20007, USA; eg239@georgetown.edu; 4 Department of Pathology, Georgetown University School of Medicine, Washington, DC 20007, USA; seema.agarwal@georgetown.edu

## Error in Figures

In the original publication [1], there were mistakes in Figures 1b and 4b as published. In Figure 1b (top panel), the mouse tumor images for Day 11 were inadvertently used for the Day 25 time point. In Figure 4b, which is a composite of three gels, lanes 9 and 10 are alternating GAPDH and T3 primers that should have been taken from Gel 3, but were mistakenly taken from Gel 1. The corrected Figure 1b and Figure 4b appear below. The three original gels for Figure 4b are now included in Supplementary Figure S5.

## Error in Supplementary Materials

With this correction, the Supplementary Materials Part has been adjusted accordingly.

**Supplementary Materials:** The following are available online at https://www.mdpi.com/2072-6694/11/10/1490/s1, Figure S1: (a) CRISPR Cas9 pLenti plasmid from ABM and Gene Targets, (b) Frameshift mutation analysis; Figure S2: (a) Frameshift mutation analysis from Next Generation Sequencing and (b) visualization of allelic mutations at CRISPR target sites; Figure S3: Scans of whole gel western blots showing molecular weight sizes of relevant proteins (indicated in arrows) for Figures 1f, 2a, 3a, 4c, 5a, 6b, 6d, 6f, and 6g; Figure S4: Densitometry analysis of western blots; Figure S5: RT-PCR gel images for Figure 4b.

The authors state that the scientific conclusions are unaffected. This correction was approved by the Academic Editor. The original publication has also been updated.

## Figures and Tables

**Figure 1:**
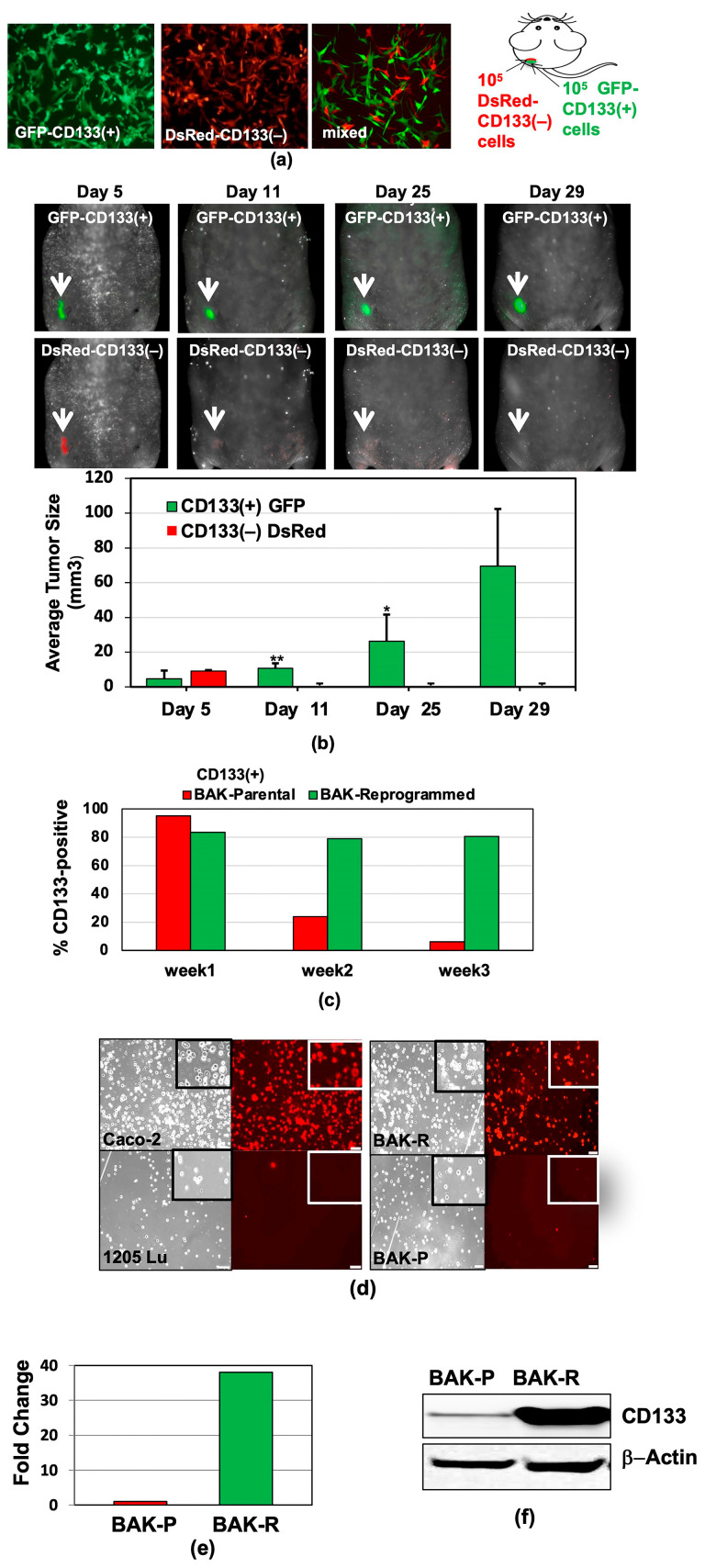
Preferential survival and growth of MACS-sorted CD133(+) cells in mixed population xenografts, and sustained CD133 expression after reprogramming. GFP-CD133(+) and DsRed-CD133(−) cells were isolated by MACS (**a**), injected into the flanks of nude mice, and tumors were visualized and measured by Maestro in vivo imaging after indicated periods of time. *p*-values of <0.05 were considered statistically significant and represented with a single asterisk, while *p* < 0.01 are labeled with two asterisks (**b**). (**c**) CD133 expression of reprogrammed BAK-R MIC and BAK-P populations assessed by immunofluorescent staining with anti-CD133-PE for 3 weeks show that BAK-R, but not BAK-P melanoma cells, exhibit sustained CD133 expression. Representative images after immunofluorescent staining of BAK-P and BAK-R cells with anti-CD133-PE ((**d**), right panels) as well as qRT-PCR (**e**) and immunoblot (**f**) analyses showing elevated CD133 expression in BAK-R compared to BAK-P cells. CD133(+) Caco-2 colon cancer cells and CD133(−) 1205Lu melanoma cells served as positive and negative controls, respectively ((**d**); left panels).

**Figure 4:**
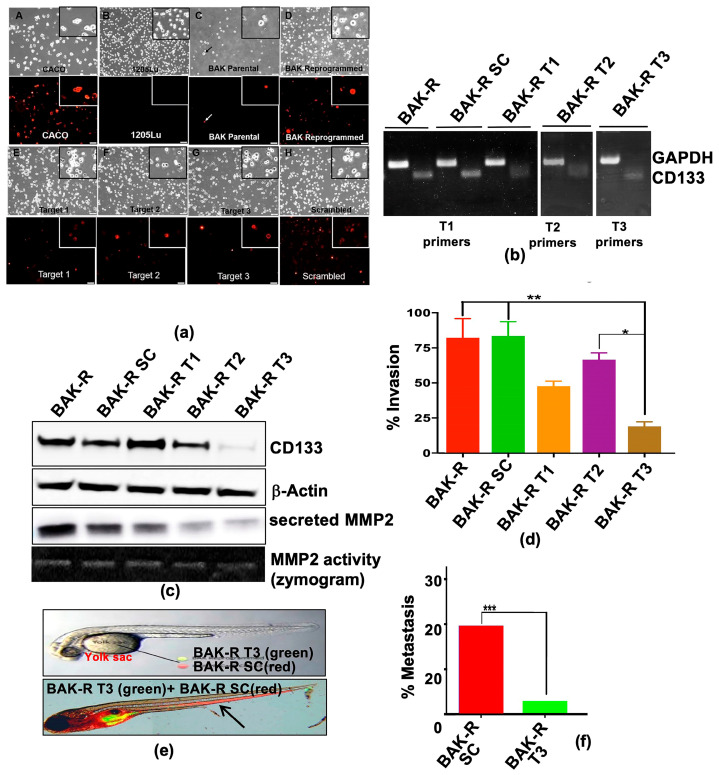
(**a**) CD133-depleted partial CRISPR-Cas9 BAK-R T3 cells show decreased CD133 expression and are less invasive and metastatic than BAK-R-SC controls. CD133 positivity was determined by immunofluorescence staining in BAK-P, BAK-R, partial CRISPR-Cas9 knockdowns using three target sgRNAs and a scrambled sgRNA control. CD133^+^ Caco-2 colon cancer cells and 1205Lu cells served as positive and negative controls, respectively. Images were taken at 10× magnification. RT-PCR (**b**) and immunoblot analysis (**c**) show depletion of CD133 RNA and protein in BAK-R T3 compared to BAK-R SC or BAK-R cells. (**d**) Transwell cell invasion assays showed decreased cell invasion in CD133-depleted BAK-R T3 cells, compared to BAK-R, control SC sgRNA, or T1 and T2 cells. (**e**) Injection of zebrafish with a 1:1 mixture of BAK-R SC (red) and BAK-R CD133 knockdown T3 cells (green) and representative images after 5 days; (**f**) quantification of % metastasis to the zebrafish tail. *, **, *** represent *p* < 0.05 and *p* < 0.01, and *p* < 0.001, respectively.
